# Improved Linearity with Polarization Coulomb Field Scattering in AlGaN/GaN Heterostructure Field-Effect Transistors

**DOI:** 10.1038/s41598-018-19510-y

**Published:** 2018-01-17

**Authors:** Peng Cui, Yuanjie Lv, Huan Liu, Aijie Cheng, Chen Fu, Zhaojun Lin

**Affiliations:** 1 School of Microelectronics, Shandong University, Jinan, 250100 China; 2 National Key Laboratory of Application Specific Integrated Circuit (ASIC), Hebei Semiconductor Research Institute, Shijiazhuang, 050051 China; 3 School of Mathematics, Shandong University, Jinan, 250100 China

## Abstract

The single-tone power of the AlGaN/GaN heterostructure field-effect transistors (HFETs) with different gate widths was measured. A distinct improvement in device linearity was observed in the sample with a larger gate width. The analysis of the variation of the parasitic source access resistance showed that, as the gate bias is increased, the polarization Coulomb field scattering can offset the increased polar optical phonon scattering and improve the device linearity. This approach is shown to be effective in improving the device linearity of AlGaN/GaN HFETs.

## Introduction

During the last decade, AlGaN/GaN heterostructure field-effect transistors (HFETs) have been extensively developed in the area of RF power electronics due to their high electron mobility and high breakdown electric field^1–3^. Device linearity is a crucial requirement for power amplifiers in wireless base stations, satellite communications, and radar applications. Linear distortion, which has hindered maximizing the advantages of AlGaN/GaN HFETs, has most recently been attracting extensive attention, given the increasingly thorough and widespread application of AlGaN/GaN HFETs in power amplifiers^4–7^.

Polarization Coulomb field (PCF) scattering, caused by the non-uniform distribution of the polarization charges at the AlGaN/GaN interface, is a particular scattering mechanism in AlGaN/GaN HFETs^8–10^. PCF scattering has been found to be capable of affecting the parasitic source access resistance and the device transconductance^10,11^, which are relevant to the device linearity. However, sufficient evidence of the effect of PCF scattering on device linearity is lacking in both experiments and theoretical studies. Previous studies have reported that PCF scattering can be changed by the material component and device structure^9,10^. This means that studying the effect of PCF scattering on device linearity may contribute to improving the linearity at the device level.

In this research, two types of AlGaN/GaN HFETs with different gate widths were fabricated. Then, the single-tone power was measured for the two samples. By analyzing the gain and input power at the 1-dB compression point, the effect of PCF scattering on the device linearity was explored.

## Results and Discussion

**Figure 1:**
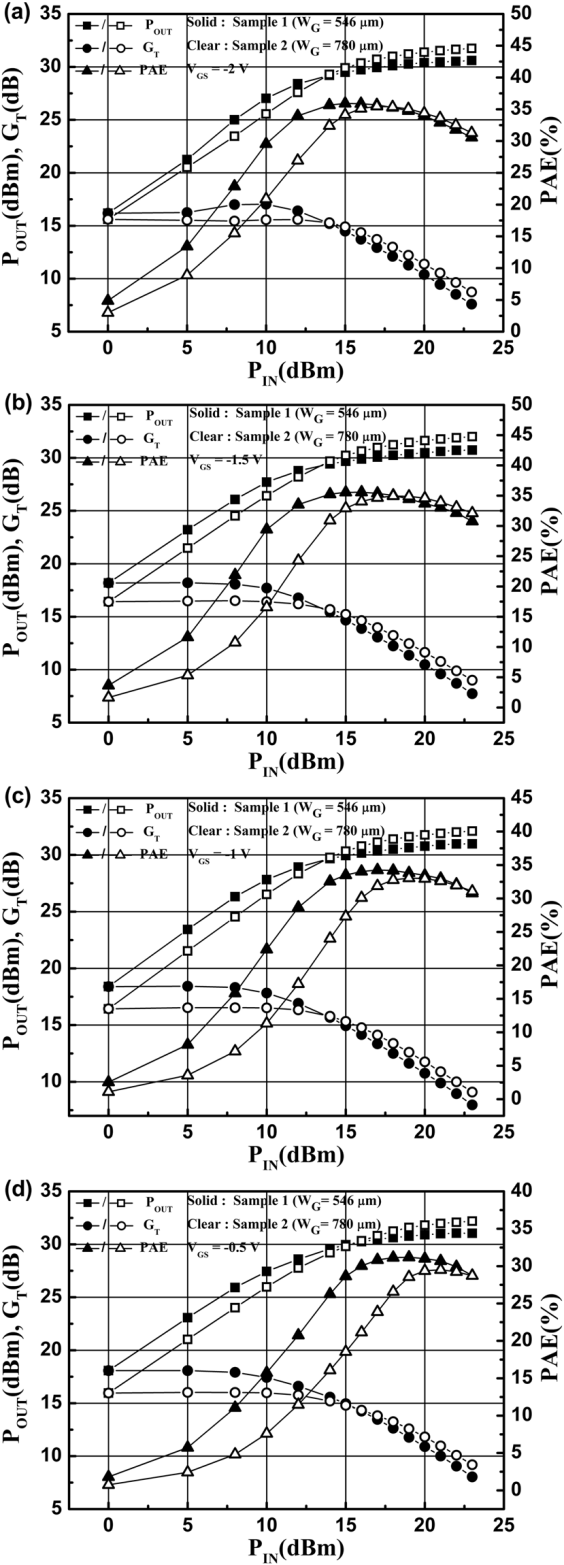
The output power *P*_OUT_, gain *G*_T_, and power added efficiency PAE as a function of the input power *P*_IN_ for the two samples with *V*_DS_ = 20 V at gate-source voltages of (**a**) −2 V, (**b**) −1.5 V, (**c**) −1 V, and (**d**) −0.5 V, respectively.

The on-wafer RF power performances were tested by using a single-tone continuous-wave signal at 2.7 GHz. At a drain voltage of 20 V, the device matching was optimized for the maximum output power. The gate biases were chosen as −2 V, −1.5 V, −1 V, and −0.5 V, respectively. The match condition was correlated with the device structure and the chosen direct current quiescent points (DCQPs). Therefore, the detailed match parameters under different DCQPs were different for two samples, as shown in Table 1. Here, Γ_S_ and Γ_L_ refer to the source matching point and the load matching point, respectively. Figure 1 shows the output power (*P*_OUT_), gain (*G*_T_), and power added efficiency (PAE) as a function of the input power (*P*_IN_) for the two samples. The *G*_T_ variation range for Sample 2 is obviously smaller than that for Sample 1. A flatter gain curve implies better device linearity. This means that Sample 2, which has a larger gate width, has better linearity. To further compare the linearity between the two samples, the input power at the 1-dB compression point *P*_IN-1dB_ was extracted from Fig. 1, as shown in Table 1. The difference in *P*_IN-1dB_ between the two samples can be written as

1 $${\rm{\Delta }}=\frac{{P}_{\mathrm{IN} \mbox{-} 1{\rm{dB}}}({\rm{Sample}}\,2)-{P}_{\mathrm{IN} \mbox{-} 1{\rm{dB}}}({\rm{Sample}}\,1)}{{P}_{\mathrm{IN} \mbox{-} 1{\rm{dB}}}({\rm{Sample}}\,1)}\times 100 \% .$$

**Table 1:** The detailed match parameters and the input power at 1-dB compression point *P*_IN-1dB_ under different DCQPs for two samples.

*V*_GS_ (V)	*V*_DS_ (V)	Sample 1 (*W*_G_ = 546 μm)				Sample 2 (*W*_G_ = 780 μm)				Δ (%)
		*I*_DS_ (A/mm)	Γ_S_	Γ_L_	*P*_IN-1dB_ (×10^−2^ W/mm)	*I*_DS_ (A/mm)	Γ_S_	Γ_L_	*P*_IN-1dB_ (×10^−2^ W/mm)	
−2	20	0.041	0.030 + *j*0.742	0.246 + *j*0.133	3.33	0.039	−0.280 + *j*0.661	0.171 + *j*0.141	4.62	38.71
−1.5	20	0.110	−0.131 + *j*0.697	0.326 + *j*0.120	2.33	0.103	−0.311 + *j*0.564	0.194 + *j*0.076	3.49	50.00
−1	20	0.165	−0.269 + *j*0.670	0.336 + *j*0.051	2.24	0.154	−0.331 + *j*0.469	0.194 + *j*0.076	5.57	148.37
−0.5	20	0.212	−0.275 + *j*0.657	0.261 + *j*0.067	2.23	0.199	−0.317 + *j*0.463	0.120 + *j*0.079	3.55	58.89

Under every fixed gate bias, the *P*_IN-1dB_ for Sample 2 is significantly larger than that for Sample 1; Δ is at least 38.71% and can reach up to 148.37% (at *V*_GS_ = −1 V).

**Figure 2:**
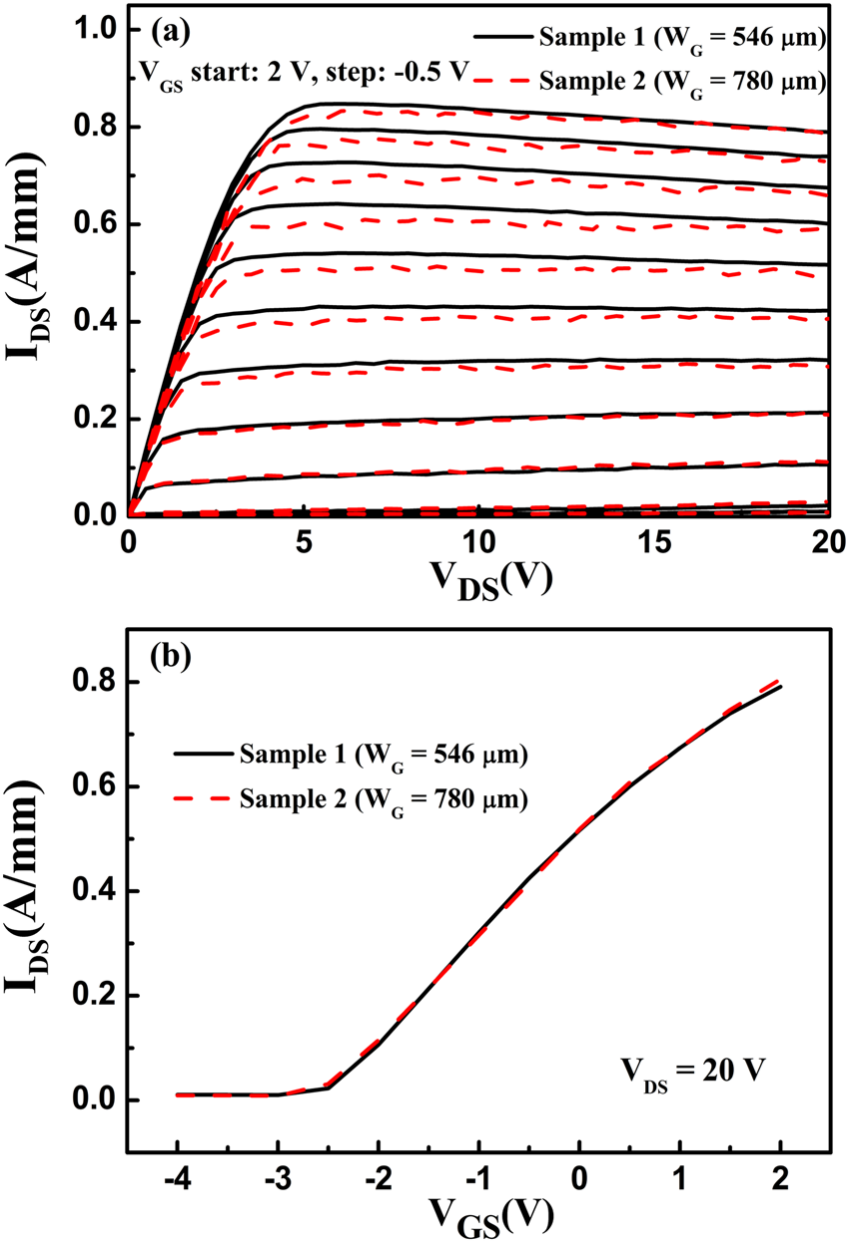
(**a**) The DC *I*-*V* characteristics and (**b**) the transfer characteristics for the two samples.

The linearity in power amplification is well known to be a complex phenomenon. The charge trapping in the surface state, gate-drain capacitance, self-heating effect, device transconductance, and parasitic source access resistance can affect the device linearity^7,10,12–16^. Because both samples were fabricated on the same material and with the same device technology, the charge trapping in the surface state and the gate-drain capacitance should be the same. The DC current-voltage (*I*-*V*) characteristics and the transfer characteristics were measured for the two samples, as shown in Fig. 2. The currents are almost the same for the two samples; therefore, the influence of the self-heating effect on the linearity of the two samples should be consistent. Because of the polarization Coulomb field scattering, the gate width can affect the parasitic source access resistance (*R*_S_) and transconductance (*g*_m_) under the unit gate width^17^. Because the ohmic contact resistance *R*_C_ (in the normalized unit “Ω·mm”) is constant, *R*_S_ here is exclusive of *R*_C_ and refers only to the gate-source channel resistance. Considering that both samples have the same device size, except for their different gate widths, the intrinsic transconductance (*g*_m0_) under the unit gate width for the two samples should be the same. An analysis of the expression *g*_m_ = 1/(1/*g*_m0_ + *R*_S_ + *R*_C_) indicates that the *R*_S_ variation can affect *g*_m_, and then influence the gain and the device linearity^7,10^. Therefore, the improved linearity can be explained by considering the variation of *R*_S_.

*R*_S_ is determined by the scattering mechanisms in the gate-source channel. The main scattering mechanisms in the gate-source channel include polar optical phonon (POP), deformation potential (DP), piezoelectric (PE), interface roughness (IFR), dislocation (DIS), and polarization Coulomb field (PCF) scatterings. Among these, the two major mechanisms are POP and PCF scatterings, which can be changed with the increase of the gate voltage.

**Figure 3:**
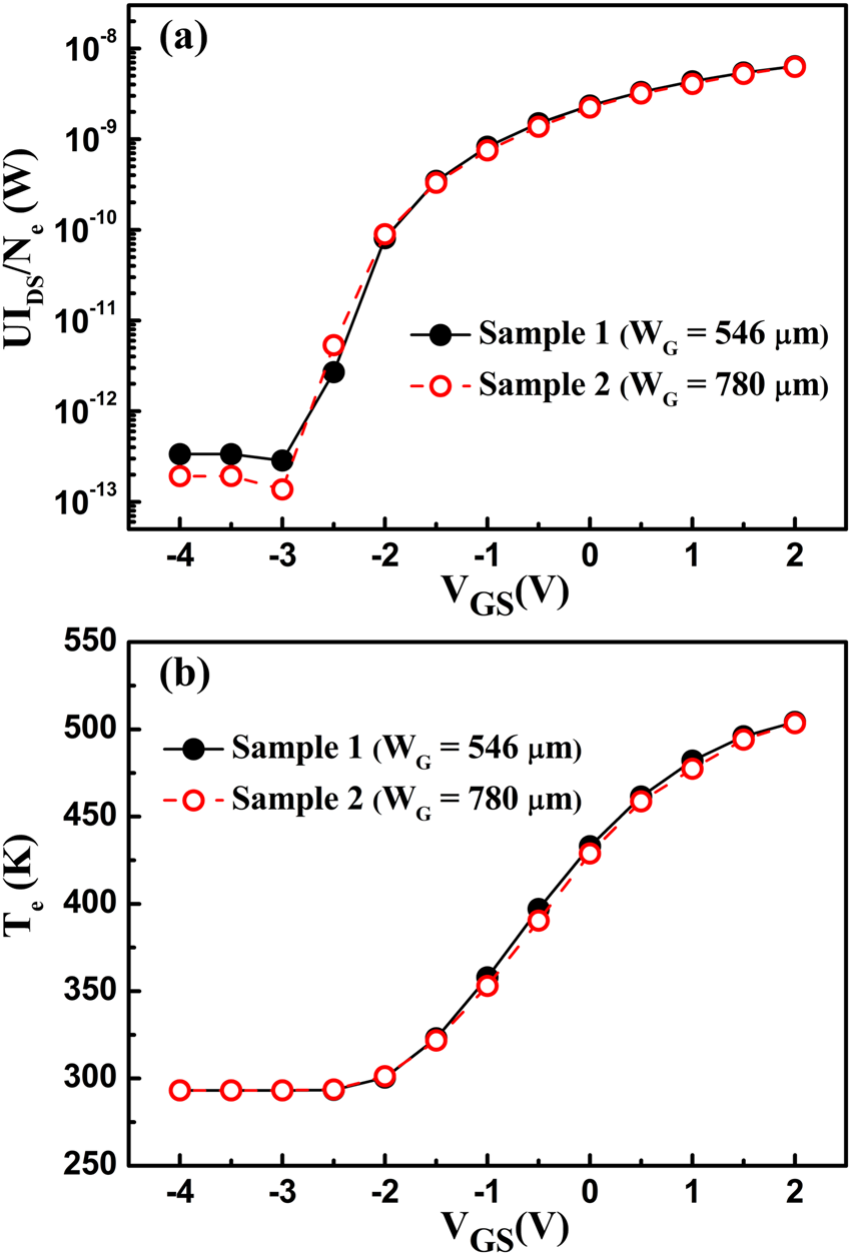
(**a**) The dissipated power per electron UI_DS_/N_*e*_ and (**b**) the electron temperature T_e_ in the gate-source channel as a function of the gate-source voltage for the two samples.

**Figure 4:**
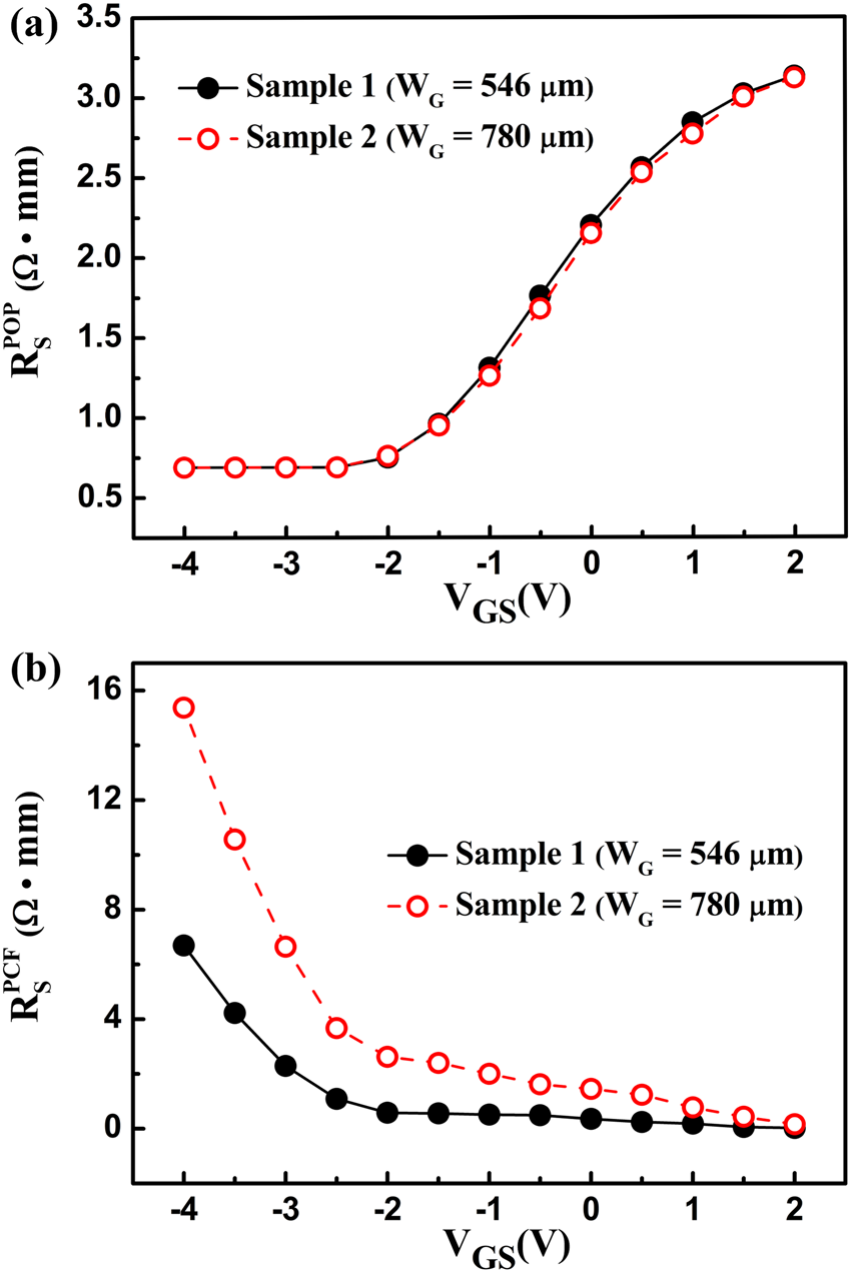
The *R*_S_ determined by (**a**) the polar optical phonon scattering ${R}_{{\rm{S}}}^{{\rm{POP}}}$ and (**b**) the polarization Coulomb field scattering ${R}_{{\rm{S}}}^{{\rm{PCF}}}$.

When the electron drift velocity is sufficiently increased, the POP and electron temperatures start to increase; the POP scattering is enhanced with the increase of the electron temperature, inducing an increase in *R*_S_^10^. For a clearer presentation, the POP scattering and the electron temperature as a function of *V*_GS_ at *V*_DS_ = 20 V can be calculated. Initially, the electron drift velocity *v*_e_ in the gate-source channel can be obtained from the *I*-*V* characteristic by applying *I*_DS_ = *n*_2D_∙*q*∙*v*_e_. With the obtained *v*_e_, the electric field *E*_GS_ in the gate-source channel can be determined by the dependence of the electron drift velocity on the electric field^18^. Then, the dissipated power per electron *UI*_DS_/*N*_*e*_ in the gate-source channel can be calculated, as shown in Fig. 3(a). Here, *U* = *E*_GS_∙*L*_GS_ is the voltage applied along the gate-source channel, *L*_GS_ is the gate-source distance, and *N*_*e*_ = *n*_2D_∙*L*_GS_∙*W*_G_ is the number of electrons in the gate-source channel. Finally, based on the relationship between the electron temperature and the dissipated power per electron^18^, the electron temperature *T*_e_ can be obtained, as shown in Fig. 3(b). As the gate bias is increased, the electron temperature is increased, and it remains at almost the same value for the two samples. This means that the influence of self-effect on *R*_S_ is the same for the two samples^17^. The *R*_S_ determined by the POP scattering ${R}_{{\rm{S}}}^{{\rm{POP}}}$ can be calculated as follows^9^

2 $${R}_{{\rm{S}}}^{{\rm{POP}}}=\frac{{L}_{{\rm{GS}}}}{{n}_{2{\rm{D}}}q{\mu }_{{\rm{POP}}}}=\frac{{L}_{{\rm{GS}}}{m}^{\ast }}{{n}_{2{\rm{D}}}{q}^{2}}\cdot \frac{1}{{\tau }_{{\rm{POP}}}}=\frac{{L}_{{\rm{GS}}}{m}^{\ast }}{{n}_{2{\rm{D}}}{q}^{2}}\cdot \frac{{e}^{2}{\omega }_{{\rm{POP}}}{m}^{\ast }{N}_{{\rm{B}}}({T}_{{\rm{e}}})G({k}_{0})}{2{\varepsilon }^{\ast }{k}_{0}{\hslash }^{2}{P}_{{\rm{POP}}}(y)},$$

where m^*^ is the electron effective mass in GaN, ɛ* = *ɛ*_0_/(1/*ɛ*_h_ − 1/*ɛ*_s_), *ɛ*_0_ is the vacuum dielectric permittivity, *ɛ*_h_ is the high-frequency dielectric constant of GaN, *ɛ*_s_ is the static dielectric constant of GaN, *y* = *πћ*^2^*n*_2D_/m^*^k_B_*T*_e_, *k*_B_ is the Boltzmann constant, *ћω*_POP_ is the POP energy, *k*_0_ = (2 m^*^(*ћω*_POP_)/*ћ*^2^)^1/2^ is the POP wave vector, *N*_B_(*T*_*e*_) = 1/exp(*ћω*_POP_/*k*_B_*T*_e_) − 1 is the Bose-Einstein function, *G*(*k*_0_) = *b*(8*b*^2^ + 9*k*_0_*b* + 3*k*_0_^2^)/(8(*k*_0_ + *b*)^3^) and *P*_POP_(*y*) = 1 + (1 + *e*^−*y*^)/*y*. As shown in Fig. 4(a), when the gate voltage is more than −2.5 V, the increased POP scattering causes *R*_S_ to increase as the gate voltage is increased.

PCF scattering originates from the non-uniform distribution of the polarization charges at the AlGaN/GaN interface^8–10^. Before the device processing or without the gate bias, the polarization charges at the AlGaN/GaN interface are uniform. On one hand, to form the ohmic contacts, Ti/Al/Ni/Au was deposited and then rapidly thermally annealed at 850 °C. During the annealing process, the ohmic contact metal atoms can diffuse into the AlGaN barrier layer and change the barrier layer strain^9,19^. On the other hand, because of the converse piezoelectric effect, the gate bias can also change the strain of the AlGaN barrier layer under the gate region^9,20^. The strain variation of the AlGaN barrier layer causes the variation of the polarization charges. Then, the distribution of the polarization charges becomes non-uniform. Compared with the uniformly distributed polarization charges, the non-uniformly distributed ones can generate an additional scattering potential, which can scatter the channel electrons. The additional polarization charges are defined as the difference between the non-uniformly distributed polarization charges and the uniformly distributed ones. After the device processing, the additional polarization charges near the ohmic contact area do not change, and their influence on the PCF scattering is constant. The additional polarization charge ∆σ under the gate region can be calculated as^10,20^:

3 $${\rm{\Delta }}{\sigma }{=}\frac{{e}_{33}^{2}}{{C}_{33}}\cdot \frac{{V}_{\text{GS}}-{V}_{{\rm{ch}}}}{{d}_{{\rm{AlGaN}}}},$$

where *e*_33_ is the piezoelectric coefficient, *C*_33_ is the elastic stiffness tensor of AlGaN, *V*_ch_ is the potential in the channel, and *d*_AlGaN_ is the thickness of the AlGaN barrier layer. As shown in (3), ∆σ is relevant to *V*_GS_. The larger ∆σ is, the stronger the PCF scattering. As *V*_GS_ is increased, ∆σ decreases and the PCF scattering weakens. The PCF scattering is stronger in the sample with a larger width^17^. Therefore, under the same gate voltage, Sample 2 has a larger PCF scattering than Sample 1. The *R*_S_ determined by the PCF scattering ${R}_{{\rm{S}}}^{{\rm{PCF}}}$ can be obtained^11^, as shown in Fig. 4(b). ${R}_{{\rm{S}}}^{{\rm{PCF}}}$ clearly shows a monotonic decline as the gate bias is increased. Because Sample 2, which has a larger width, has a stronger PCF scattering, its ${R}_{{\rm{S}}}^{{\rm{PCF}}}$ is larger compared with Sample 1.

**Figure 5:**
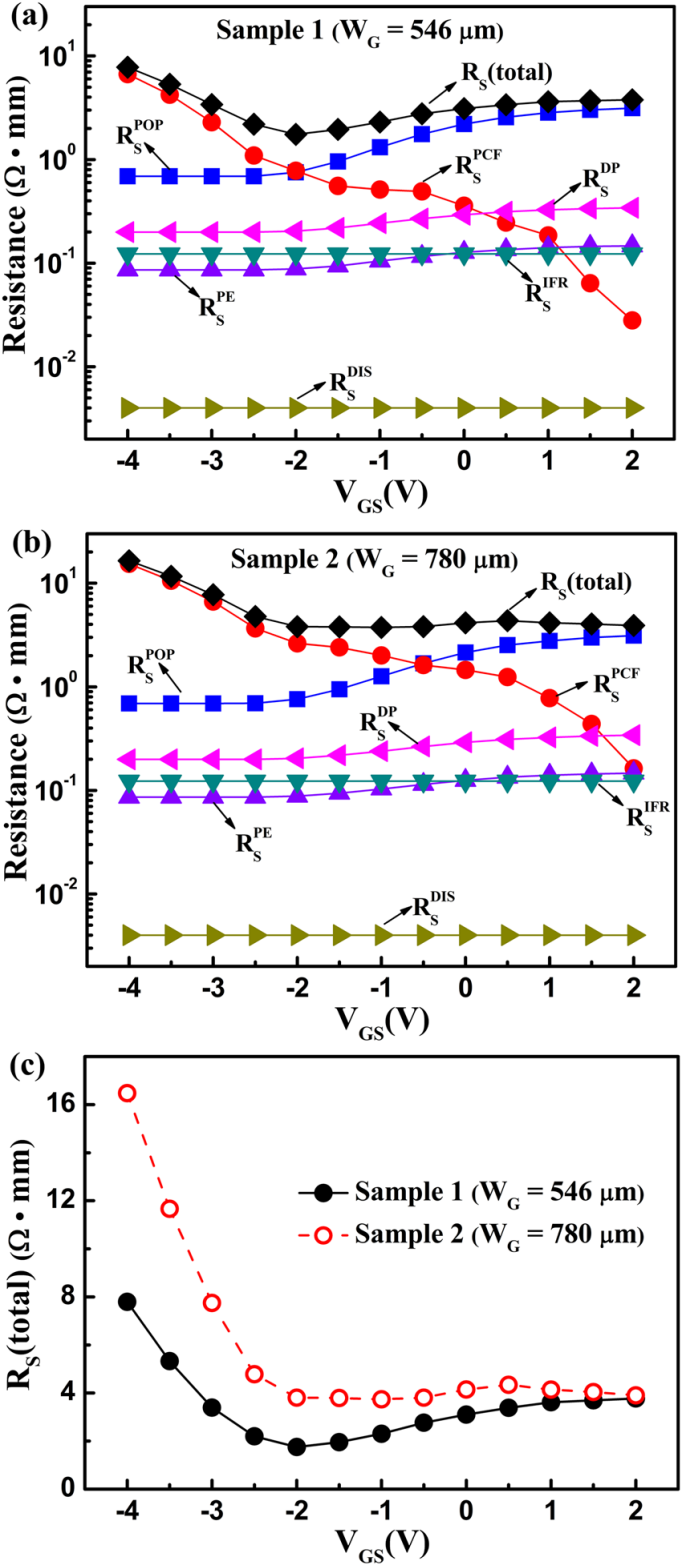
(**a**) The *R*_S_ determined by the polar optical phonon scattering ${R}_{{\rm{S}}}^{{\rm{POP}}}$, polarization Coulomb field scattering ${R}_{{\rm{S}}}^{{\rm{PCF}}}$, deformation potential scattering ${R}_{{\rm{S}}}^{{\rm{DP}}}$, piezoelectric scattering ${R}_{{\rm{S}}}^{{\rm{PE}}}$, interface roughness scattering ${R}_{{\rm{S}}}^{{\rm{IFR}}}$, and dislocation scattering ${R}_{{\rm{S}}}^{{\rm{DIS}}}$; the total gate-source resistance values *R*_S_ (total) for (**a**) Sample 1 and (**b**) Sample 2, and (**c**) the total gate-source resistance values *R*_S_ (total) as a function of the gate-source voltage for the two samples.

As the gate bias is increased, the POP scattering is increased and the PCF scattering is decreased; together, these determine the variation of *R*_S_. The decreased PCF scattering can effectively offset the increased POP scattering, decrease the variation of *R*_S_, and then improve the linearity. This causes Sample 2, which has a larger PCF scattering, to have better linearity. The *R*_S_ values for different scattering mechanisms were calculated^11,21^, as shown in Fig. 5(a) and (b). The POP, DP, and PE scatterings are enhanced with the increased gate bias, leading to the increase in *R*_S_. Among these three mechanisms, POP scattering is the major one. Conversely, PCF scattering is the only mechanism that is decreased with the increased gate bias. The decreased PCF scattering can offset the increased scatterings, causing the *R*_S_ value to have a small variation. For a clear comparison, Fig. 5(c) shows the total *R*_S_ for the two samples. As shown in Fig. 2(b), the threshold voltage for the two samples is −2.5 V, therefore the *V*_GS_ in the range of −2.5 V to 2 V is effective. During the effective gate bias range, the *R*_S_ for Sample 2 is flatter than that for Sample 1, which means that Sample 2 has a smaller *R*_S_ variation. Based on *g*_m_ = 1/(1/*g*_m0_ + *R*_S_ + *R*_C_), a smaller *R*_S_ variation implies a smaller *g*_m_ variation and better device linearity. Hence, Sample 2 shows better linearity.

In addition, when the gate bias is more negative, the PCF scattering is stronger than the POP scattering, and *R*_S_ is decreased with the increased gate bias. As the gate bias is increased, the POP scattering is rapidly increased with the increase of the electron temperature. When *V*_GS_ = −1 V was chosen as the DCQP, the offset effect between the PCF and the POP scattering was the most suitable for the power output. Therefore, when *V*_GS_ = −1 V, the offset range for POP and PCF scattering is the largest, and the improvement in linearity is most apparent (corresponding to Δ = 148.37%). This further confirmed that PCF scattering exerts a vital influence on the device linearity by affecting *R*_S_.

## Conclusion

The single-tone power of the AlGaN/GaN HFETs with different gate widths was measured, and the improvement in linearity was determined. The results indicate that PCF scattering can offset the increased POP scattering as the gate bias is increased, as well as enhance the linearity of the devices. Thus, the approach is effective in improving the device linearity of AlGaN/GaN HFETs.

## Methods

### Sample fabrication

The AlGaN/GaN heterostructure was grown by molecular beam epitaxy (MBE). The epitaxial structure was grown on a sapphire substrate consisting of, from the bottom to the top, a 40-nm-thick AlN buffer layer, a 2-μm-thick GaN channel layer, a 1-nm-thick AlN interlayer, and a 20-nm-thick Al_0.2_Ga_0.8_N barrier layer. The Hall measurement yielded a two-dimensional electron gas (2DEG) sheet electron density (*n*_2D_) of 8 × 10^12^ cm^−2^ and an electron mobility (*μ*) of 2000 cm^2^/V∙s at room temperature. The device fabrication started with mesa isolation, which was formed by inductively coupled plasma reactive ion etching (ICP-RIE) with the use of a BCl_3_/Cl_2_ gas mixture. Ti/Al/Ni/Au (300/1500/500/600 Å) was evaporated and annealed at 850 °C for 30 s in nitrogen atmosphere to form the drain and source ohmic contacts. The space between the drain and source ohmic contacts was 6 μm. Transmission-line matrix measurements showed that the specific contact resistivity of the ohmic contacts was 2 × 10^−5^ Ω·cm^2^. Ni/Au (600/2000 Å) two-finger gate with 1-μm gate length (*L*_G_) was fabricated and located in the middle of the drain and source ohmic contacts. Finally, the devices were passivated by using a 100-nm-thick SiN layer deposited by PECVD. Devices with gate width (*W*_G_) of 546 μm (2 × 273 μm) and 780 μm (2 × 390 μm) were marked as Samples 1 and 2, respectively.

### Measurements

The on–wafer RF power performance of uncooled devices were tested by using a Maury load-pull system. The *I-V* characteristics were measured with the use of an Agilent B1500A semiconductor parameter analyzer.

## References

[1] Maassen D (2017). 70W GaN-HEMT Ku-band power amplifier in MIC technology. IEEE Trans. Microw. Theory Techn..

[2] Paine BM, Polmanter SR, Ng VT, Kubota NT, Ignacio CR (2017). Fast-pulsed characterizations of RF GaN HEMTs during wearout. IEEE Trans. Device Mater. Rel..

[3] Ishida H (2005). A High-power RF switch IC using AlGaN/GaN HFETs with single-stage configuration. IEEE Trans. Electron Devices..

[4] Nagy W, Brown J, Borges R, Singhal S (2003). Linearity characteristics of microwave-power GaN HEMTs. IEEE Trans. Microw. Theory Techn..

[5] Islam SS, Mehdi Anwar AF (2002). Temperature-dependent nonlinearities in GaN/AlGaN HEMTs. IEEE Trans. Electron Devices..

[6] Gao T (2015). Improved linearity in AlGaN/GaN metal-insulator-semiconductor high electron mobility transistors with nonlinear polarization dielectric. Appl. Phys. Lett..

[7] Palacios T (2006). Use of double-channel heterostructures to improve the access resistance and linearity in GaN-based HEMTs. IEEE Trans. Electron Devices..

[8] Zhao J (2007). Electron mobility related to scattering caused by the strain variation of AlGaN barrier layer in strained AlGaN/GaN heterostructures. Appl. Phys. Lett..

[9] Luan C (2014). Theoretical model of the polarization Coulomb field scattering in strained AlGaN/AlN/GaN heterostructure field-effect transistors. J. Appl. Phys..

[10] Yang M (2016). Effect of polarization Coulomb field scattering on parasitic source access resistance and extrinsic transconductance in AlGaN/GaN heterostructure FETs. IEEE Trans. Electron Devices..

[11] Yang M (2016). Study of source access resistance at direct current quiescent points for AlGaN/GaN heterostructure field-effect transistors. J. Appl. Phys..

[12] Wang W (2015). Analysis of the contact resistance in amorphous InGaZnO thin film transistors. Appl. Phys. Lett..

[13] Wang W (2013). Modified transmission line model for bottom-contact organic transistors. IEEE Electron Device Lett..

[14] Bag, A. *et al*. Effect of longitudinal electric field and self heating of channel on linearity and gain of AlGaN/GaN HEMT on sapphire (0001). *In Proc. 2014 IEEE Students’ Technology Symposium, Kharagpur, India*, 393–395 (2014).

[15] Wu YF (2004). 30-W/mm GaN HEMTs by field plate optimization. IEEE Electron Device Lett..

[16] Huang J (2010). Linearity characteristics of field-plated AlGaN/GaN high electron mobility transistors for microwave applications. Jpa. J. Appl. Phys..

[17] Yang M (2016). Study of gate width influence on extrinsic transconductance in AlGaN/GaN heterostructure field-effect transistors with polarization Coulomb field scattering. IEEE Trans. Electron Devices..

[18] Matulionis A (2003). Hot-phonon temperature and lifetime in a biased Al_*x*_Ga_1-*x*_N/Gan channel estimated from noise analysis. Phys. Rev. B..

[19] Luan C (2012). Influence of the side-Ohmic contact processing on the polarization Coulomb field scattering in AlGaN/AlN/GaN heterostructure field-effect Transistors. Appl. Phys. Lett..

[20] Anwar AFM, Webster RT, Smith KV (2006). Bias induced strain in AlGaN/GaN heterojunction field effect transistors and its implications. Appl. Phys. Lett..

[21] Cui P (2017). Influence of different gate biases and gate lengths on parasitic source access resistance in AlGaN/GaN heterostructure FETs. IEEE Trans. Electron Devices..

